# Performance evaluation of a new sponge-based moving bed biofilm reactor for the removal of pharmaceutical pollutants from real wastewater

**DOI:** 10.1038/s41598-024-64442-5

**Published:** 2024-06-20

**Authors:** Zohreh Chalipa, Majid Hosseinzadeh, Mohammad Reza Nikoo

**Affiliations:** 1 School of Civil Engineering, Iran University of Science and Technology, Narmak, Tehran 1684613114 Iran; 2 Department of Civil and Architectural Engineering, Sultan Qaboos University, Muscat, Oman

**Keywords:** Pharmaceutical, Ibuprofen, Moving bed biofilm reactor, Wastewater, Response surface methodology, Biological techniques, Biotechnology, Environmental sciences

## Abstract

Pharmaceutical pollutants, a group of emerging contaminants, have attracted outstanding attention in recent years, and their removal from aquatic environments has been addressed. In the current study, a new sponge-based moving bed biofilm reactor (MBBR) was developed to remove chemical oxygen demand (COD) and the pharmaceutical compound Ibuprofen (IBU). A 30-L pilot scale MBBR was constructed, which was continuously fed from the effluent of the first clarifier of the Southern Tehran wastewater treatment plant. The controlled operational parameters were pH in the natural range, Dissolved Oxygen of 1.5–2 mg/L, average suspended mixed liquor suspended solids (MLSS), and mixed liquor volatile suspended solids (MLVSS) of 1.68 ± 0.1 g/L and 1.48 ± 0.1 g/L, respectively. The effect of hydraulic retention time (HRT) (5 h, 10 h, 15 h), filling ratio (10%, 20%, 30%), and initial IBU concentration (2 mg/L, 5 mg/L, 10 mg/L) on removal efficiencies was assessed. The findings of this study revealed a COD removal efficiency ranging from 48.9 to 96.7%, with the best removal efficiency observed at an HRT of 10 h, a filling ratio of 20%, and an initial IBU concentration of 2 mg/L. Simultaneously, the IBU removal rate ranged from 25 to 92.7%, with the highest removal efficiency observed under the same HRT and filling ratio, albeit with an initial IBU concentration of 5 mg/L. An extension of HRT from 5 to 10 h significantly improved both COD and IBU removal. However, further extension from 10 to 15 h slightly enhanced the removal efficiency of COD and IBU, and even in some cases, removal efficiency decreased. Based on the obtained results, 20% of the filling ratio was chosen as the optimum state. Increasing the initial concentration of IBU from 2 to 5 mg/L generally improved COD and IBU removal, whereas an increase from 5 to 10 mg/L caused a decline in COD and IBU removal. This study also optimized the reactor’s efficiency for COD and IBU removal by using response surface methodology (RSM) with independent variables of HRT, filling ratio, and initial IBU concentration. In this regard, the quadratic model was found to be significant. Utilizing the central composite design (CCD), the optimal operating parameters at an HRT of 10 h, a filling ratio of 21%, and an initial IBU concentration of 3 mg/L were pinpointed, achieving the highest COD and IBU removal efficiencies. The present study demonstrated that sponge-based MBBR stands out as a promising technology for COD and IBU removal.

## Introduction

In recent years, pharmaceutical compounds have been ubiquitously detected in the aquatic environment^1,2^. Several critical factors have contributed to their increase in wastewater and treated water: (1) Advancements in technology have enabled researchers to detect the concentrations of these pollutants within the ng/L to μg/L range. Achieving extraction at these low levels necessitates utilizing reliable sample preparation techniques, thereby revealing the extent of their presence in the environment^3–5^. (2) The dramatic increase in the consumption of these compounds worldwide has contributed to their prevalence in the environment^5,6^. (3) Improper disposal of unused or expired medications, often flushed down the toilet or poured down the drain^7^. (4) Some pharmaceuticals resist complete breakdown by organisms and are excreted into the environment, making it an urgent issue that requires immediate attention^8^. Pharmaceutically-active compounds (PhACs), a specific subset of micro-pollutants, are also classified as an emerging group of contaminants. Given that conventional treatment plants are not tailored to remove these substances, treated effluent discharge from wastewater treatment plants (WWTPs) is a significant means by which pharmaceutical compounds are introduced into the aquatic environment. In fact, due to their low concentration, relatively high polarity, and non-biodegradability, it is challenging to remove them. In addition to the seasonal conditions causing fluctuations in the treatment efficiency of pharmaceuticals, there is a lack of stringent regulations in most WWTPs for monitoring these compounds^9–12^. As a result, some of them will persist in the effluent and end up in the environment, polluting the surface and groundwater.

Ibuprofen (IBU), a popular non-steroidal anti-inflammatory drug (NSAID), is one of the most highly prescribed PhACs and the most salable over-the-counter medicine worldwide, which humans use to alleviate pain, inflammation, and fever^13,14^. It is included in the Essential Medicines List 2010 published by the World Health Organization (WHO). The IBU consumption rate in several countries varies from 14.2 to 345 tones/year^15^. It should be noted that as much as 85% of consumed IBU is excreted by urine and feces without undergoing metabolism^14^. Consequently, IBU consistently has been identified in the influent of municipal wastewater treatment plants. Furthermore, as conventional treatment methods are insufficient in eliminating IBU due to its high polarity, hydrophilic nature, therefore less sorption to sludge, and water solubility, IBU has often been found in municipal WWTPs' discharge and has arrived in other water bodies^12,16–18^. For example, Yang et al.^18^ have reported an overall IBU removal of 56–64% in a conventional WWTP located in Spain and a 72% removal rate in Greece. Several studies have investigated the IBU concentration in various water bodies. Luo et al.^19^ have reported the average IBU concentration in surface waters was 0.98 µg/L in Canada, 8.0 µg/L in France, 1417 µg/L in China, 1.0–67 µg/L in Greece, 15–414 µg/L in Korea, and 5.0–280 µg/L in Taiwan. The study conducted by Marsik et al.^20^ identified IBU as the most abundant drug in a basin located in the Czech Republic, with a peak concentration of 3210 ng/L. IBU has also been observed in groundwaters because of water leaching, and it is recognized as one of the emerging contaminants posing significant risks to the human health and environment^21–23^. Lapworth et al.^23^ have documented that the mean concentration of IBU based on studies conducted in 14 countries across Europe, the Middle East, North America, and Asia is 1.5 µg/L. IBU was found in surface water and groundwater samples in Cameroon at concentrations of 516 ng/L and 276 ng/L, respectively^24^.

In spite of the low concentrations, IBU can have detrimental impacts on the environment and human health mainly due to its ability to be entered and accumulated in the food chain through discharge of effluent and the utilization of treated wastewater and sludge in agricultural practices^18,25^. IBU, as a pharmaceutical compound, may persist in water bodies and soil, impacting aquatic organisms and terrestrial wildlife. It can disrupt physiological processes, alter behavior, and impair reproduction in various species. Additionally, IBU can bioaccumulate in organisms, leading to magnification of its effects up the food chain. Ultimately, these impacts can contribute to biodiversity loss and ecosystem degradation^26^. Numerous toxicity studies have been carried out to examine the harmful effects of IBU on aquatic organisms^27–30^. Xia et al.^31^ have explored the IBU effect on the early stages of D. rerio. Zebrafish embryos by exposing them to 5, 50, and 500 µg/L IBU from 6 up to 120 h post-fertilization (hpf). The outcomes they obtained indicated that the IBU significantly reduced the hatch rate at 55 hpf, and it had a substantial impact on the ability of zebrafish embryos to move and was potentially neurotoxic^31^. In another study, Hodkovicova et al.^32^ claimed that exposure to IBU caused several adverse effects on the kidneys and the liver of freshwater fish Oncorhynchus mykiss. Moreover, exposure to uncontrolled low-dose IBU in the first and second trimesters of pregnancy can increase the likelihood of genital abnormalities in new-born boys and be harmful to cell proliferation in human embryonic cells^33,34^. Also, to this date, there is no regulation or limit set for IBU in the environment^13,35^. Therefore, the proper elimination of IBU from the environment should be taken into serious consideration. Improving conventional wastewater treatment processes such as activated sludge through optimization or upgrading is of great importance to achieve a further reduction of IBU in WWTP's discharge.

Advanced treatment techniques, such as membrane filtration^36,37^, membrane bioreactor (MBR)^38,39^, activated carbon adsorption^40^, ozonation^41^, and advanced oxidation processes (AOPs)^42,43^, have been developed to remove IBU effectively. However, these technologies can be costly to operate and may present challenges. Membrane-based methods incur high investment costs and can suffer from concentrated residue formation and membrane fouling. Additionally, ozonation and advanced oxidation processes (AOPs) require significant energy consumption and equipment costs, and may produce toxic byproducts^44^. In this regard, attached-growth processes are an auspicious alternative to suspended-growth-based activated sludge processes^44^. The Moving bed biofilm reactor (MBBR), an attached-growth-based technique introduced in Norway in 1988, involves adding suspended carriers to the reactor, allowing the biomass to adhere and grow^45–47^. Doing so not only eases the proliferation of microorganisms with a slow growth rate, which is crucial for eliminating certain micropollutants, but also provides an aerobic and anaerobic environment within the carriers, enhancing nutrient removal and a wide range of micropollutants^44,48^. By benefiting from attached and suspended growth simultaneously, MBBR offers several advantages, such as improving treatment capacity, requiring a small floor area, eliminating the need for sludge reflux or backwashing, having low head loss and power consumption, and being highly resistant to changes in temperature and sewage composition^49^. For these benefits, MBBR is considered a promising wastewater treatment technology and has been utilized in over 1200 WWTPs across at least 50 countries^50^. Although MBBR has been shown to be an effective process in eliminating conventional pollutants (e.g., organic matter and nutrients)^51,52^, there is a lack of research on removing PhACs, particularly IBU, in MBBR. Even so, some studies have demonstrated MBBR with plastic-based carriers as an inefficient technique for IBU removal from wastewater. In a study conducted by Fatehifar et al.^10^, the elimination of IBU from synthetic wastewater was investigated using MBBR with Kaldnes media. The results revealed that IBU removal ranged from 11.33 to 37.33% at HRT = 10 h and 0 to 35.10% at HRT = 5 h^10^. Thus, it is necessary to investigate the enhancement of MBBR performance using other carrier types and fill the knowledge gap with further research. Additionally, previous studies have explored the various parameters’ impact individually on IBU removal through MBBR, such as HRT and filling ratio, and mostly conducted their experiments on lab-scale setups using synthetic wastewater^44,53^. However, to the best of our knowledge, a comprehensive investigation that simultaneously examines the combined impact of these parameters on IBU removal in an MBBR system remains lacking. Additionally, this study enhanced the applicability of its research findings by conducting experiments using real wastewater in a pilot-scale MBBR in continuous mode.

Based on the provided information, previous studies predominantly employed plastic biofilm carriers, conducted experiments in lab-scale reactors using synthetic wastewater, and primarily focused on examining individual parameters’ effects. Therefore, this study endeavors to fill these gaps by:

1. Evaluating the efficacy of a novel sponge-based MBBR in removing IBU from real wastewater.
2. Assessing the impact of various key factors, including HRT, filling ratio, and initial IBU concentration simultaneously, on the reactor's performance.
3. Modeling the operational and process conditions of continuous pilot-scale MBBR using the central composite design (CCD) of response surface methodology (RSM).

## Materials and methods

### Chemical and materials

IBU, with a purity of 98%, was acquired from Raha Pharmaceuticals, a highly regarded pharmaceutical manufacturer based in Isfahan, Iran. All other chemicals, including NaOH, H2SO4, HCl, methanol, acetic acid, and acetonitrile (HPLC grade), were purchased from Merk, Germany.

### MBBR characteristics

**Figure 1:**
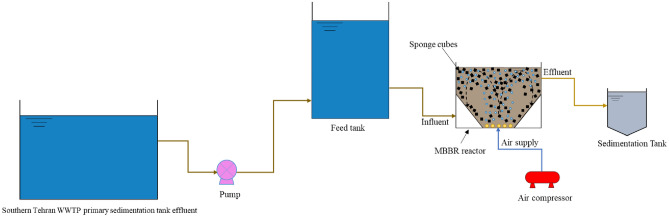
Schematic view of the studied MBBR system.

**Figure 2:**
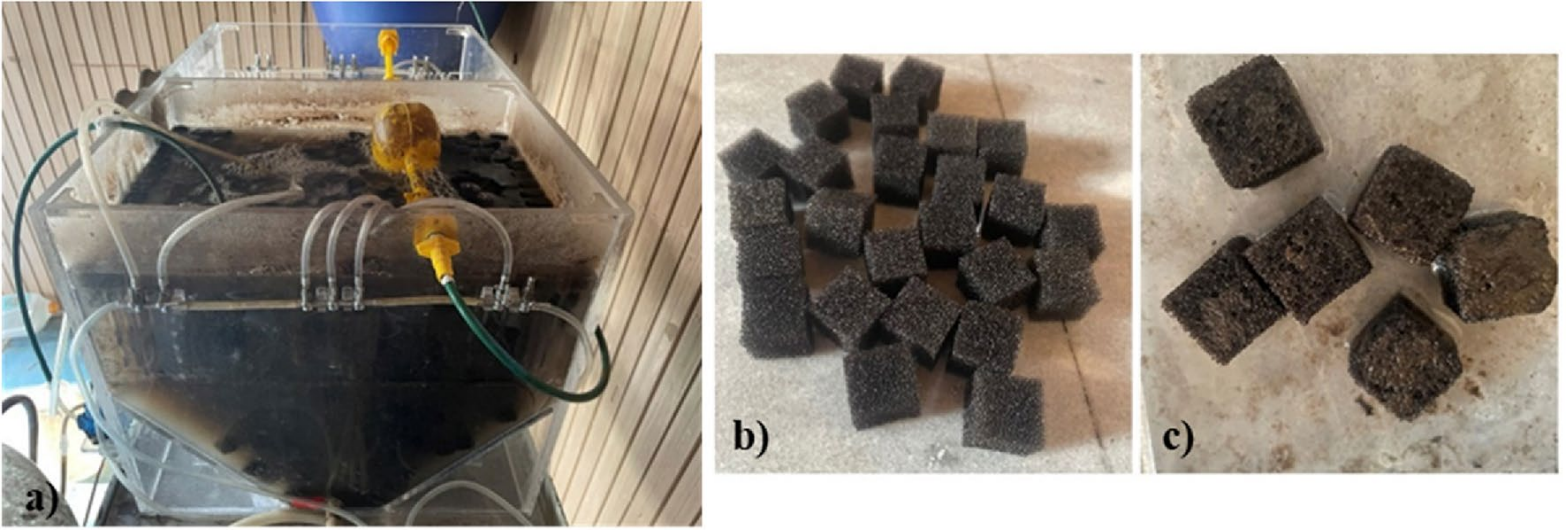
(**a**) MBBR reactor, Sponge-based carriers (**b**) Clean carriers (**c**) Carriers with attached biofilm.

A pilot-scale MBBR system with an effective volume of 30 L was employed. In this reactor, two diagonal plates were used to enhance the rotation of carriers (Figs. 1 and 2a). Polyurethane sponge cubes with a density of 12 kg/m^3^, average weight of 0.124 g, specific surface area of 1589.8 m^2^/kg, and dimension of 2 cm × 2 cm × 2 cm were utilized as biofilm carriers (Fig. 2b). Five air diffusers were installed at the reactor’s end, and the aeration of the MBBR was adjusted in the range of 1.5–2 mg/L to provide sufficient oxygen supply in the reactor and proper circulation of carriers. A schematic view of the studied MBBR system is shown in Fig. 1, and the MBBR reactor, clean carriers, and carriers with attached growth are presented in Fig. 2a–c.

### Experimental set-up

To conduct experiments, as shown in Fig. 1, a feed tank with 100 L volume, a pilot-scale MBBR, and a 4 L sedimentation tank were set up at the Southern Tehran WWTP, the greatest treatment plant in the Middle East. The feed tank was continuously fed with the effluent of the primary clarifier of the Southern Tehran WWTP with an average COD value of 274 ± 69 mg/L, and an average IBU concentration of 23.5 µg/L. The sedimentation tank was positioned at the MBBR reactor outlet for the clarification of effluent and separation of sludge.

The experimental set-up of this study was comprised of two steps. The first was creating a conducive environment for the biofilm to grow on the carriers, which is called acclimatization. To initiate the process, approximately two-thirds of the reactor was filled with active sludge from the return sludge of the secondary clarifier of Southern Tehran WWTP (MLSS = 3160 mg/L, COD = 4440 mg/L). During this step, the reactor was operated in a continuous mode with an HRT set at 24 h. Accordingly, the reactor had a flow rate of 1.25 L/h; also, no IBU was added in this step. This step continued for 21 days until stable COD removal was achieved, and the formation of attached biomass on the carriers was ensured. In the second step, IBU was added to the feed, and the reactor’s performance in IBU and COD removal was investigated under three HRTs (5 h, 10 h, and 15 h), three filling ratios (10%, 20%, and 30%), and three initial IBU concentrations (2 mg/L, 5 mg/L, and 10 mg/L). Table 1 demonstrates the operational conditions within the reactor.

**Table 1:** The operational conditions within the reactor.

Parameter	pH	DO	Temperature	Suspended MLSS	Suspended MLVSS	COD
Average amount	7–8	1.5–2 mg/L	23.5 ± 4 °C	1.68 ± 0.1 g/L	1.48 ± 0.1 g/L	274 ± 69 mg/L

### Sample preparation

In all experiments, 30 mL of sample was extracted from the influent and effluent of the reactor. The COD was analyzed immediately, and the remaining sample was acidified to pH 2 with 1 M HCl and stored in a dark glass container in the fridge at 4 °C. The high-performance liquid chromatography (HPLC) test for measuring IBU was conducted within a week. Prior to analysis, the samples were left to reach room temperature. For COD tests, 2 mL of samples were utilized, while 20 µL was employed for HPLC tests. To measure the attached biomass to the sponges, sludge contained within the sponge cubes was collected by hand squeezing the cubes and rinsing the squeezed cubes with ultrapure water, as previously described by Zhang et al.^54^.

### Analytical method

**Figure 3:**
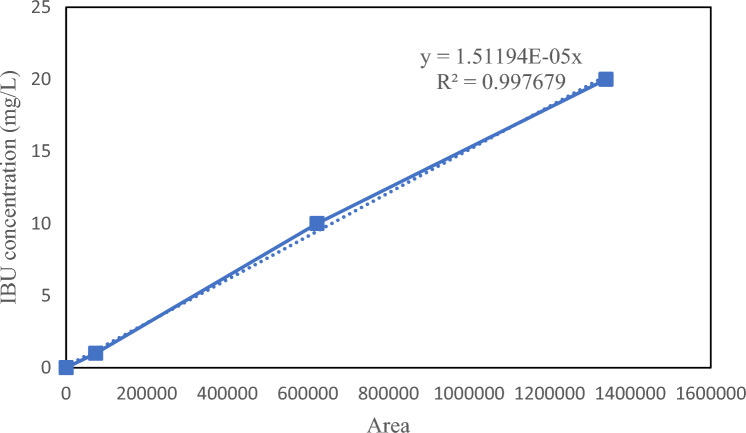
Standard curve of IBU measurement.

The influent and effluent COD analysis, the measurement of mixed liquor suspended solids (MLSS), and mixed liquor volatile suspended solids (MLVSS) concentration were conducted according to Standard Methods^55^ by using filter paper (MN 640 w), scale (RADWAG PS 510.R1), oven (Memmert), kiln (Linn), COD reactor (MN NANOCOLOR VARIO C2), and Spectrophotometer (MN NANOCOLOR UV/VIS II). DO meter (Hach HQ40 d), pH meter (Hach HQ440 d), and stirrer (IKA RH basic2) were also used during experiments. IBU measurement was carried out by HPLC–UV spectroscopy (smart line model with KNAUER-PINNCLE PCX). The analytical column was Adamas C18-X-Bond (250 × 4.6 mm^2^, 5 µm). The mobile phase was acetic acid solution with a concentration of 6.9 mmol/L adjusted to pH 6 (by NaOH) and 35% v/v acetonitrile with a 1 mL/min flow rate. Acetonitrile was chosen as phase A and acetonitrile/6.9 mmol/L acetic acid at pH 6 (40:60%, v/v) as phase B. 20 µL of the sample was filtered through polyvinylidene fluoride (PVDF) syringe filters (AXIVA, 0.2 µm filtration rating, 13 mm diameter) and then injected into the HPLC. IBU peaked at 4 min, with UV detection occurring at a wavelength of 220 nm. Standard curve of IBU measurement is shown in Fig. 3.

### Optimization of COD and IBU removal

The optimum conditions for removing COD and IBU were determined through the utilization of a central composite design (CCD) within the framework of response surface methodology (RSM), using the Design-Expert software (Stat- Ease Inc., Minneapolis, MN, USA, version 11.0). This method integrates mathematical and statistical methods to determine the relative significance of the operating factors on COD and IBU removal^56^. In this study, CCD design was developed based on three independent variables, including HRT, filling ratio, and initial IBU concentration. Table 2 displays the experimental conditions according to the factorial design. On the basis of the factorial design of the MBBR, 20 runs were designed. Then, the results of these runs were fitted into a quadratic model, Eq. (1)^57^. Nonetheless, to validate the results and thoroughly investigate the impact of all parameters, all 27 experiments were carried out.

1 $$Y={\beta }_{0}+\sum_{i=1}^{k}{\beta }_{i}{x}_{i}+\sum_{i=1}^{k}{\beta }_{ii}{{x}_{i}}^{2}+\sum_{i<j}^{k}\sum {\beta }_{ij}{x}_{i}{x}_{j}+e$$

where Y is the predicted response (COD and IBU removal efficiency), k is the number of variables, x represents independent variables, β_0_ is the constant coefficient, β_i_, β_ii_, and β_ij_ are the coefficients of linear, quadratic, and interaction terms, respectively, and e is the random error.

**Table 2:** Independent variables and their levels employed in the response surface design.

Factors	Symbol	Coded level		
		− 1	0	1
HRT (h)	A	5	10	15
Filling ratio (%)	B	10	20	30
Initial IBU concentration (mg/L)	C	2	5	10

To assess the validity of the proposed model, diagnostic checks using analysis of variance (ANOVA) with a 95% confidence interval was conducted.

## Results and discussion

### Impact of HRT on MBBR performance

#### COD removal rate

**Figure 4:**
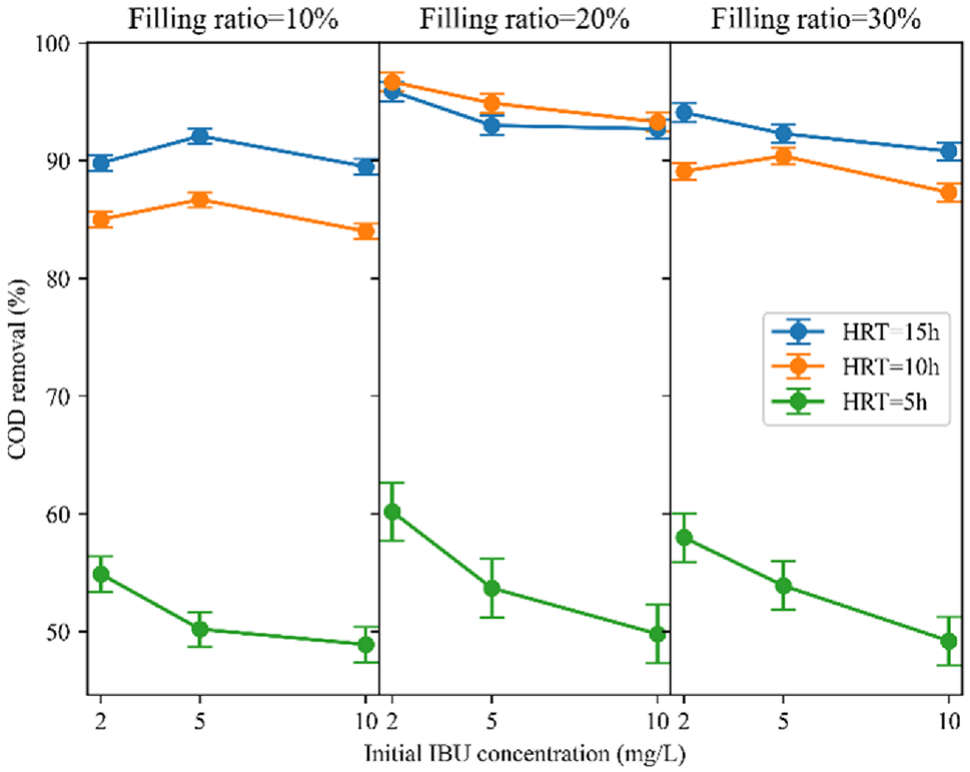
HRT impact on COD removal at different filling ratios.

In this study, it was observed that in all three filling ratios, the removal of COD was significantly lower for an HRT of 5 h compared to HRT of 10 and 15 h, as shown in Fig. 4. The higher removal rates were observed in longer HRTs can be attributed to the extended contact time between the carriers and the effluent. This extended contact time allows for microbial growth and activity within the reactor, enabling sufficient biodegradation of organic matter and consequently resulting in enhanced removal efficiency^58^. Moreover, at a shorter HRT of 5 h, there is limited substrate availability due to the rapid flow-through of wastewater. This leads to incomplete COD degradation as the microbial community may not have sufficient time to utilize the available substrates fully^59^. However, the reactor demonstrated nearly similar removal rates for HRTs of 10 h and 15 h, indicating that microorganisms effectively removed organic matter during the initial 10 h but showed no notable removal during the subsequent 5 h. For example, in HRT of 15 h and 10% filling ratio, COD removal is 4.8%, 5.4%, and 5.5% higher than HRT of 10 h for initial IBU concentrations of 2 mg/L, 5 mg/L, and 10 mg/L, respectively. This result is consistent with that of Majid et al.^60^, which achieved a slight difference of 3% in COD removal in HRTs of 8 and 12 h in an MBBR reactor with K3 Kaldnes carriers. Another study also demonstrated that extending the HRT from 8 to 12 h resulted in an increase in COD removal efficiency from 80 to 82% for ring form carriers and from 84 to 86% for Kaldnes-3 carriers when the DO concentration was 4 mg/L^61^. Nevertheless, at a 20% filling ratio, the reactor exhibited slightly better performance in a 10 h retention time compared to 15 h. This can be clarified by the food-to-microorganism (F/M) ratio, which was 0.65 g COD/g MLVSS d at the HRT of 15 h, while at the HRT of 10 h, it was 0.83 g COD/g MLVSS d, indicating that the supplied feed was more suitable for microorganism growth in the latter scenario^53^. Jiang et al.^53^ observed the highest COD removal efficiency at an 18 h HRT compared to 6, 12, and 24 h, attributed to the optimal F/M ratio achieved at this HRT duration. The Fig. 8a illustrates the average COD removal percentages for HRTs of 5, 10, and 15 h, which are 53.2%, 89.7%, and 92.2%, respectively. Considering the negligible difference in COD removal rate between 10 and 15 h, an HRT of 10 h is chosen as the optimal duration for COD removal. Selecting a shorter HRT causes the system to employ the maximum hydraulic capacity and reduces the energy required for aeration^60^.

#### IBU removal rate

**Figure 5:**
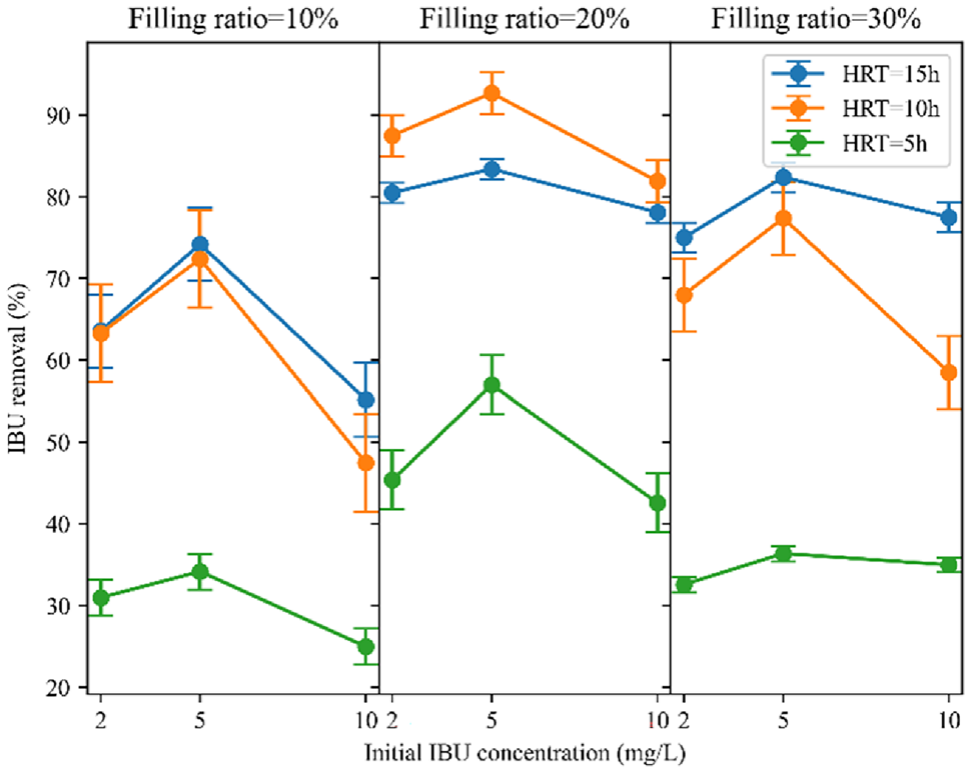
HRT impact on IBU removal at different filling ratios.

Similarly, the removal of IBU was significantly reduced during a HRT of 5 h, primarily due to the limited contact time between the pollutant and microorganisms mentioned earlier. Figure 5 demonstrates that a longer retention time of 10 h led to a better IBU removal rate, indicating that the half-life of IBU necessitates a longer reaction time. Yu et al.^62^ previously reported moderate biodegradability of IBU, achieving a 77% degradation of IBU within a 4-day period through an immobilized cell process. When the filling ratio is either 10% or 30%, extending the HRT from 10 to 15 h enhances the efficiency of IBU removal in the reactors. Only at a filling ratio of 20% does the IBU removal rate at 10 h HRT surpass that at 15 h. Similar to COD removal, this occurred due to adequate food supply for microorganisms at 10 h HRT (F/M = 0.83 g COD/g MLVSS d), which closely aligned with the value of 0.91 g COD/g MLVSS d as reported by Jiang et al.^53^. It should be noted that biodegradation was the primary pathway for IBU removal. Other factors, such as sorption to biomass contribute less to IBU removal compared to biodegradation due to its faster nature. However, sorption onto biomass can extend residence time, enhancing removal via biodegradation^53^. Jiang (2016) documented IBU removal rates of 97.8%, 98.4%, 97.1%, and 92.6% at HRTs of 24, 18, 12, and 6 h, respectively, in a sponge-based MBBR. It can be observed that IBU removal rates increased slightly with HRTs exceeding 12 h; moreover, a decrease was noted as the HRT increased from 18 to 24 h^63^. From Fig. 8c, the average IBU removal rates were 37.7%, 72.1%, and 74.4% for HRTs of 5, 10, and 15 h, respectively.

### Impact of filling ratio on MBBR performance

#### COD removal rate

**Figure 6:**
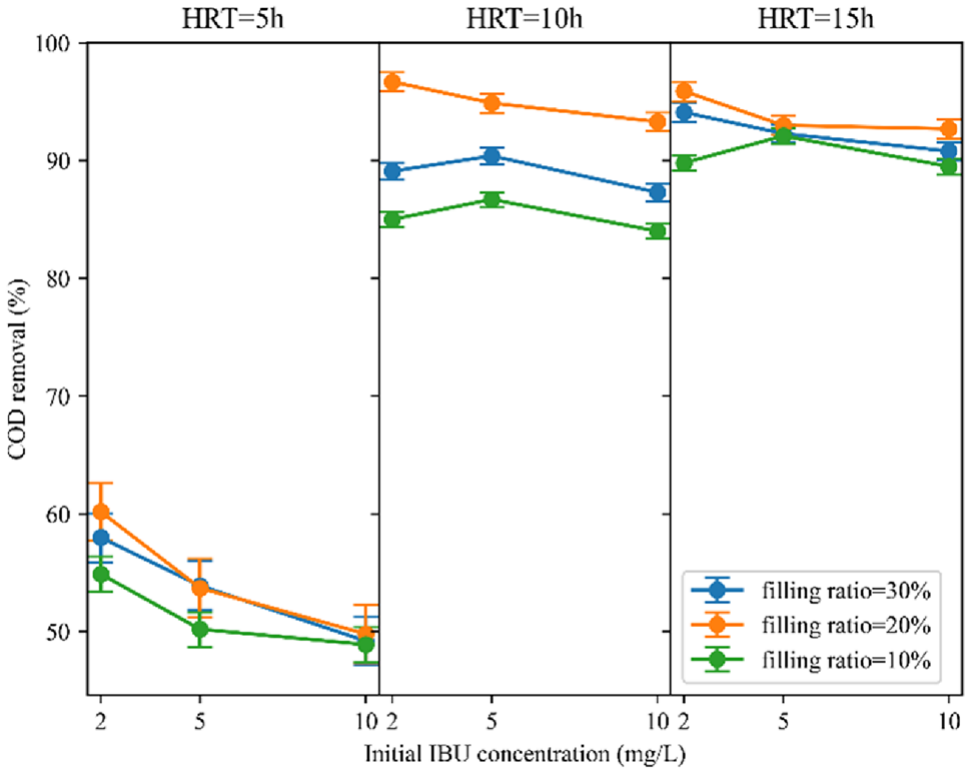
Filling ratio impact on COD removal at different HRTs.

Figure 6 illustrates COD removal efficiencies in various filling ratios. The reactor performed well at all three filling ratios. Nonetheless, the reactor performed slightly better in a 20% filling ratio than in 10% and 30%. Increasing the filling ratio from 10 to 20% improved the COD removal, which could be understandable from increasing the biofilm biomass of the system due to the greater number of carriers. Although elevating the filling ratio from 20 to 30% increased biofilm biomass within the reactor, it adversely affected the substrates’ transfer of the biomass into the sponges. As a result, less COD removal was observed. A study employing polyethylene carriers at filling ratios of 20%, 30%, 40%, and 50% demonstrated elevated COD removal rates with increasing filling ratio^64^. Similarly, in another investigation targeting coking wastewater using polyethylene carriers, COD removal rates rose as the filling ratio escalated from 20 to 50%, but declined at 60%^65^. This trend may stem from the carriers' material composition, indicating that plastic carriers facilitate sufficient circulation at higher filling ratios than sponge-based carriers. However, it is advised that the filling ratio should not surpass 70% to ensure optimal mixing properties of the carriers^52^. Studies employing sponge-based carriers did not exceed filling ratios of 30%^54,66^. Moreover, the F/M ratios in the 10%, 20%, and 30% filling ratios were 1.4, 1, and 1.05 g COD/g MLVSS d, respectively. A F/M ratio of 1 g COD/g MLVSS d led to improved COD removal due to sufficient food supply for the quantity of microorganisms present. Average COD removal in 10%, 20%, and 30% filling ratios were 75.7%, 81.1%, and 78.3%, respectively (Fig. 8b). It is evident that the average COD removal across the three filling ratios was marginally different. Nevertheless, considering the average removal efficiencies, the reactor achieved the highest COD removal in a filling ratio of 20%. Likewise, a previous study has reported that the reactor achieved the highest removal efficiency of Total Organic Carbon (TOC) when it was filled with 20% sponge carriers^54^.

#### IBU removal rate

**Figure 7:**
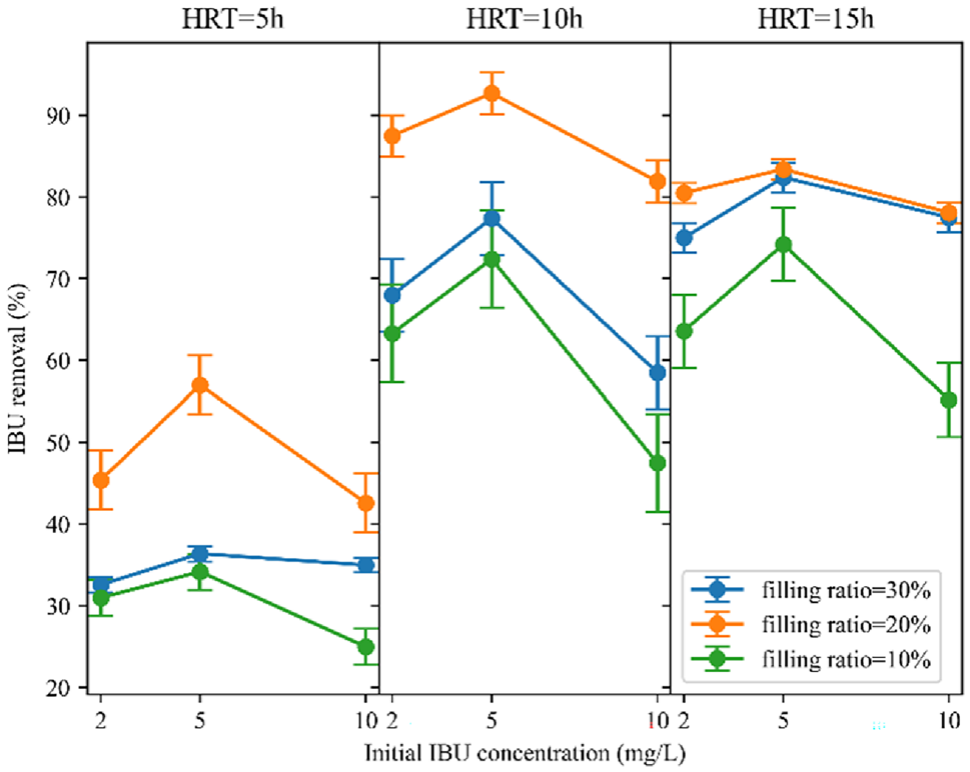
Filling ratio impact on IBU removal at different HRTs.

**Figure 8:**
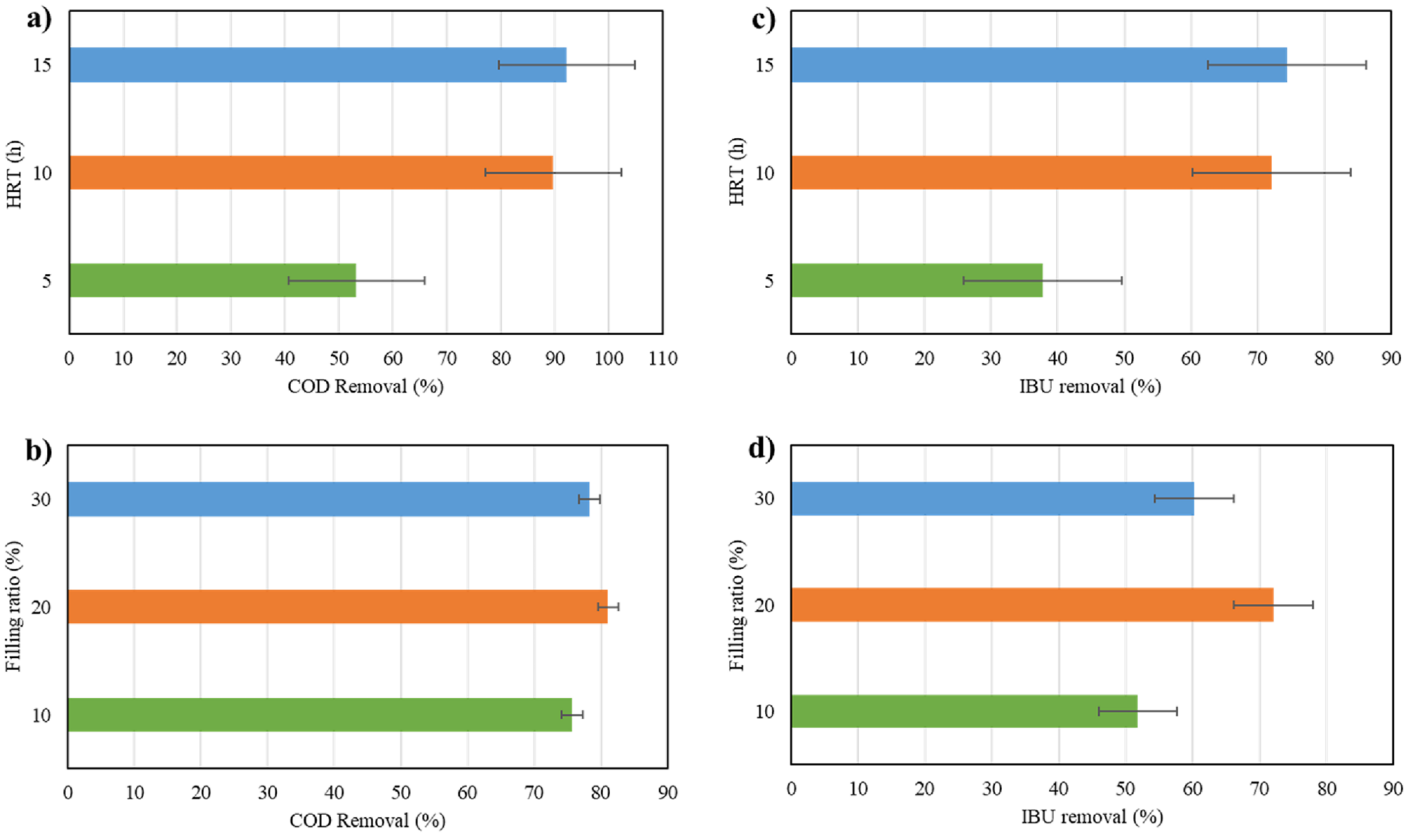
(**a**) Average COD removal at different HRTs, (**b**) Average COD removal at different filling ratios, (**c**) Average IBU removal at different HRTs and (**d**) Average IBU removal at different filling ratios.

Figure 7 provides clear evidence that a 10% filling ratio has consistently demonstrated inferior performance compared to 20% and 30% in all situations. The decline in IBU removal efficiency in the 10% filling ratio can be attributed to less available microorganisms as well as carriers’ rapid circulation. More specifically, it was observed that as the number of carriers in the reactor decreased, their circulation became more rapid. This rapid movement resulted in substantial collisions among them, resulting in the detachment and loss of attached microorganisms from the carriers. Consequently, the reactor became less effective in eliminating IBU. The reactor filled with 30% of carriers has shown medium removal rates because of floating slowly and rotating unevenly of carriers within the reactor due to their greater abundance, which in turn affects the transfer of substrate into the carriers^54^. In other words, the slower rotation of carriers in the reactor leads to the formation of a dense layer of biomass around the carrier surface, impeding the penetration of dissolved oxygen (DO) and substrate into the carriers. The optimal performance of the reactor in IBU removal can be seen by utilizing a 20% filling ratio with sponges. This filling ratio ensures the most suitable circulation for carriers, thereby causing improved performance. Similarly, Luo et al.^44^ omitted the 30% filling ratio in their experiments because of inadequate and uneven carrier circulation, and observed higher IBU removal rates at a 20% filling ratio compared to 10%. To be clearer, in 20% filling ratio, the carriers rotated uniformly, facilitating the prevention of the formation of excess biomass on their surface, as well as reducing the collision of carriers and loss of microorganisms. The attached biomass benefited from an adequate supply of substrate and DO, leading to enhanced reactor efficiency. The average attached MLSS for 10%, 20%, and 30% filling ratios was 0.57 g/L, 0.78 g/L, and 0.92 g/L, respectively. Figure 8d shows the average IBU removal in 10%, 20%, and 30% filling ratios, which are 51.8%, 72.1%, and 60.3%, respectively.

### Impact of initial IBU concentration on MBBR performance

#### COD removal rate

**Figure 9:**
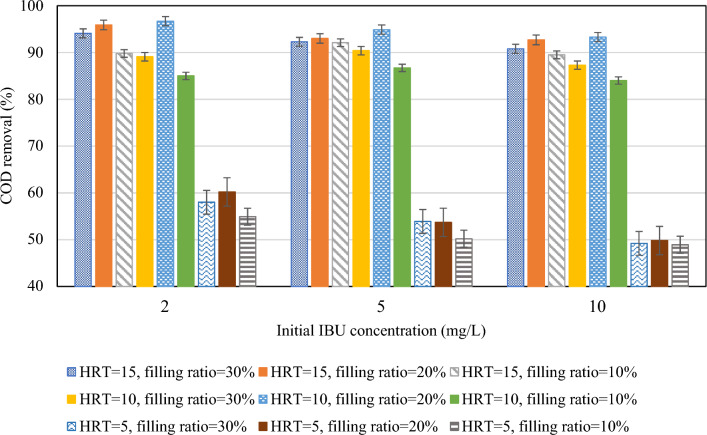
Initial IBU concentration impact on COD removal.

The initial IBU concentration effect on COD removal is depicted in Fig. 9. This experiment was done to realize whether the IBU is toxic for microorganisms and influences COD removal. With the increment of IBU concentration from 2 to 5 mg/L, and then from 5 to 10 mg/L, in six conditions, including HRT = 15 h and filling ratio = 30% and 20%, HRT = 10 h and filling ratio = 20%, and HRT = 5 h and filling ratio = 30%, 20% and 10% the COD removal decreased to only a limited extent. However, in the remaining conditions, COD removal initially increased with the rise in IBU concentration from 2 to 5 mg/L, which means that IBU did not exhibit a toxic impact; it may even stimulate microbial growth and diversity^48^. In addition, COD removal efficiency decreased with the increase of IBU concentration from 5 to 10 mg/L in these conditions, indicating that microbial diversity may decrease when IBU concentration exceeds the tolerance of microorganisms^48^. The results revealed that the maximum decline in COD removal, amounting to 10.4%, was observed where the HRT was 5 h and the filling ratio was 20%. In this condition, increasing the IBU concentration from 2 to 10 mg/L resulted in a decrease in COD removal from 60.2 to 49.8%. Based on these findings, it can be deduced that IBU did not have a significant negative effect on COD removal, even at concentrations much higher than environmentally relevant concentrations. Fatehifar et al.^10^ similarly found no significant toxic effect of IBU on COD removal with the same IBU amount.

#### IBU removal rate

**Figure 10:**
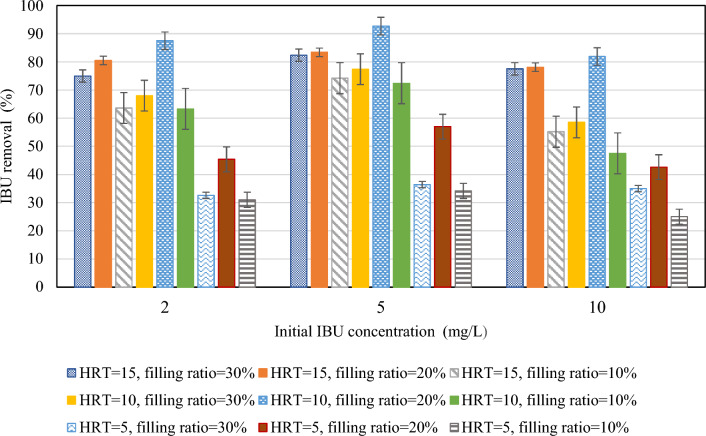
Initial IBU concentration impact on IBU removal.

Figure 10 illustrates the influence of the initial IBU concentration on IBU removal. As depicted in the figure, the removal of IBU increased as the initial concentration rose from 2 to 5 mg/L with minimum amount of 2.9 for the HRT = 15 h and filling ratio of 20%, and maximum amount of 11.6 for the HRT = 5 h and filling ratio of 20%. This can be attributed to the higher accessibility of IBU for microorganisms, leading to improved digestion. Earlier studies have also reported a decline in the removal rate of pollutants when they are present in lower concentrations^10^. However, when the IBU concentration was increased from 5 to 10 mg/L, the reactor exhibited lower IBU removal efficiency. The IBU removal efficiencies decreased from 82.4 to 77.5%, 83.4 to 78.1%, 74.2 to 55.2%, 77.4 to 58.5%, 92.7 to 81.9%, 72.4 to 47.5%, 36.4 to 35%, 57 to 42.6%, and 34.2 to 25% for HRT = 15 h and filling ratio = 30%, 20% and 10%, HRT = 10 h and filling ratio = 30%, 20% and 10%, and HRT = 5 h and filling ratio = 30%, 20% and 10%, respectively. Despite the increased availability, the concentration of 10 mg/L surpassed the capacity and tolerance of the microorganisms. Regarding metabolic pathways, bacteria capable of biodegrading IBU present potential mechanisms for its breakdown, with hydroxylation serving as a primary pathway. Different microorganisms demonstrate varied degradation routes for IBU, encompassing hydroxylation of side chains and aromatic rings^13^. To optimize efficiency, further investigations into microbial community dynamics are recommended.

### Statistical data analysis and optimization

In this study, the CCD model was employed to assess the interactive effect of factors for optimizing the COD and IBU removal from municipal wastewater. In this regard, Design-Expert was used for analysis of variance (ANOVA), and the experimental results for COD and IBU removal under various conditions were utilized as input data (Table 3), allowing us to establish the appropriate model and identify the optimal conditions. The correlation between the response variables and the independent variable has been expressed in the form of equations (Eqs. 2 and 3).

2 $$\begin{aligned} COD removal \left( \% \right) & = 93.50 + 19.32A + 1.23B - 2.18C + 0.2750AB \\ & \quad + 1.36AC - 0.7519BC - 19.80A^{2} - 4.60B^{2} + 2.55C^{2} \\ \end{aligned}$$

3 $$\begin{aligned} IBU removal \left( \% \right) & = 92 + 17.73A + 5.48B - 1.21C + 3.14AB \\ & \quad + 0.3648AC + 2.93BC - 20.56A^{2} - 15.86B^{2} - 6.14C^{2} \\ \end{aligned}$$

where A is HRT, B is the filling ratio, and C is the initial IBU concentration.

**Table 3:** Experimental conditions and responses obtained by RSM optimization.

Run	Factor 1	Factor 2	Factor 3	Response 1	Response 2
	A: HRT (h)	B: Filling ratio (%)	C: Initial IBU concentration (mg/L)	COD removal (%)	IBU removal (%)
1	5	20	5	53.7	57
2	10	30	5	90.4	77.4
3	10	20	5	94.9	92.7
4	5	30	2	58	32.6
5	10	20	2	96.7	87.5
6	5	10	10	48.9	25
7	10	20	5	94.9	92.7
8	5	10	2	54.9	31
9	15	30	2	94.1	75
10	10	20	5	94.9	92.7
11	5	30	10	49.2	35
12	10	20	5	94.9	92.7
13	10	20	5	94.9	92.7
14	15	10	2	89.8	63.6
15	15	20	5	93	83.4
16	15	30	10	90.8	80.5
17	10	20	5	94.9	92.7
18	15	10	10	89.5	55.2
19	10	20	10	93.3	81.9
20	10	10	5	86.7	72.4

The statistical significance of the factors and their interactions at various probability levels are shown in Table 4. As it is evident, overall, the quadratic model is statistically meaningful in predicting the experimental values of COD and IBU removal with a *p*-value < 0.0001. It should be noted that *p*-value < 0.05 indicates that the model terms hold significance at confidence levels of 95% or higher, while values greater than 0.10 represent that the model terms are not significant^67–69^. According to Table 4, for COD removal, model terms of A, B, C, AC, A^2^, B^2^, and C2, and for IBU removal, terms of A, B, AB, BC, A^2^, B^2^, and C^2^ have *p*-values < 0.05, indicating that they contribute positively to the model. Conversely, the remaining terms are associated with *p*-values > 0.05, suggesting that they have a negative impact on the model and warrant further investigation. Additionally, the model F-values of 558.14 and 136.01 also imply that the model is significant, and there is only a 0.01% chance that this large model value could occur due to noise.

**Table 4:** ANOVA table for the analysis of variance of the response surface quadratic model.

Source	% COD removal						% IBU removal					
	Sum of squares	df	Mean square	F-value	*p*-value		Sum of squares	df	Mean square	F-value	*p*-value	
Model	6150.41	9	683.38	558.14	< 0.0001	Significant	10,419.19	9	1157.69	136.01	< 0.0001	Significant
A-HRT	3720.33	1	3720.33	3038.55	< 0.0001		3133.23	1	3133.23	368.11	< 0.0001	
B-filling ratio	15.14	1	15.14	12.37	0.0056		298.99	1	298.99	35.13	0.0001	
C-Initial IBU concentration	47.52	1	47.52	38.81	< 0.0001		14.64	1	14.64	1.72	0.219	
AB	0.605	1	0.605	0.4941	0.4981		78.75	1	78.75	9.25	0.0124	
AC	14.94	1	14.94	12.2	0.0058		1.08	1	1.08	0.1267	0.7293	
BC	4.58	1	4.58	3.74	0.0819		69.43	1	69.43	8.16	0.0171	
A^2^	1078.61	1	1078.61	880.94	< 0.0001		1162.36	1	1162.36	136.56	< 0.0001	
B^2^	58.31	1	58.31	47.62	< 0.0001		691.65	1	691.65	81.26	< 0.0001	
C^2^	15.45	1	15.45	12.62	0.0052		89.59	1	89.59	10.53	0.0088	
Residual	12.24	10	1.22				85.12	10	8.51			
Lack of Fit	12.24	5	2.45				85.12	5	17.02			
Pure Error	0	5	0				0	5	0			
Cor Total	6162.65	19					10,504.31	19				

To validate the model’s goodness of fit, the values of coefficient of determination: R^2^, adjusted R^2^, and predicted R^2^ were determined and found to be 0.998, 0.9962, and 0.9908 for COD removal, and 0.9919, 0.9846, and 0.9357 for IBU removal, respectively. The overall appropriateness of a model prediction is generally explained by R^2^, which measures the total variation of predicted or model values from the mean^70,71^. In this study, model exhibits high predictive accuracy as the R^2^ values of both COD and IBU removal approach 1.0. However, the assessment of model prediction efficiency should not rely solely on R^2^ because R^2^ tends to increase with the addition of more terms to the model, regardless of their statistical significance. Instead, it is essential to compare the R^2^ value with the adjusted R^2^, which considers the number of factors in the experiment. The adjusted R^2^ often decreases when statistically insignificant variables are added to the model^72,73^. A marginal difference between R^2^ and adjusted R^2^ for COD and IBU removal suggests that the non-significant terms have not been included in the model. Moreover, as predicted R^2^ does not have a difference of more than 0.2 with adjusted R^2^, it is clear that the quadratic model proved to be valid for the optimization study. It's important to highlight that RSM operates within a limited experimental domain, hindering its ability to develop models across a wide range of conditions. Additionally, it relies on a second-order polynomial model for modeling, which may not adequately capture systems with curvature^74^.

### Simultaneous effect of various parameters

**Figure 11:**
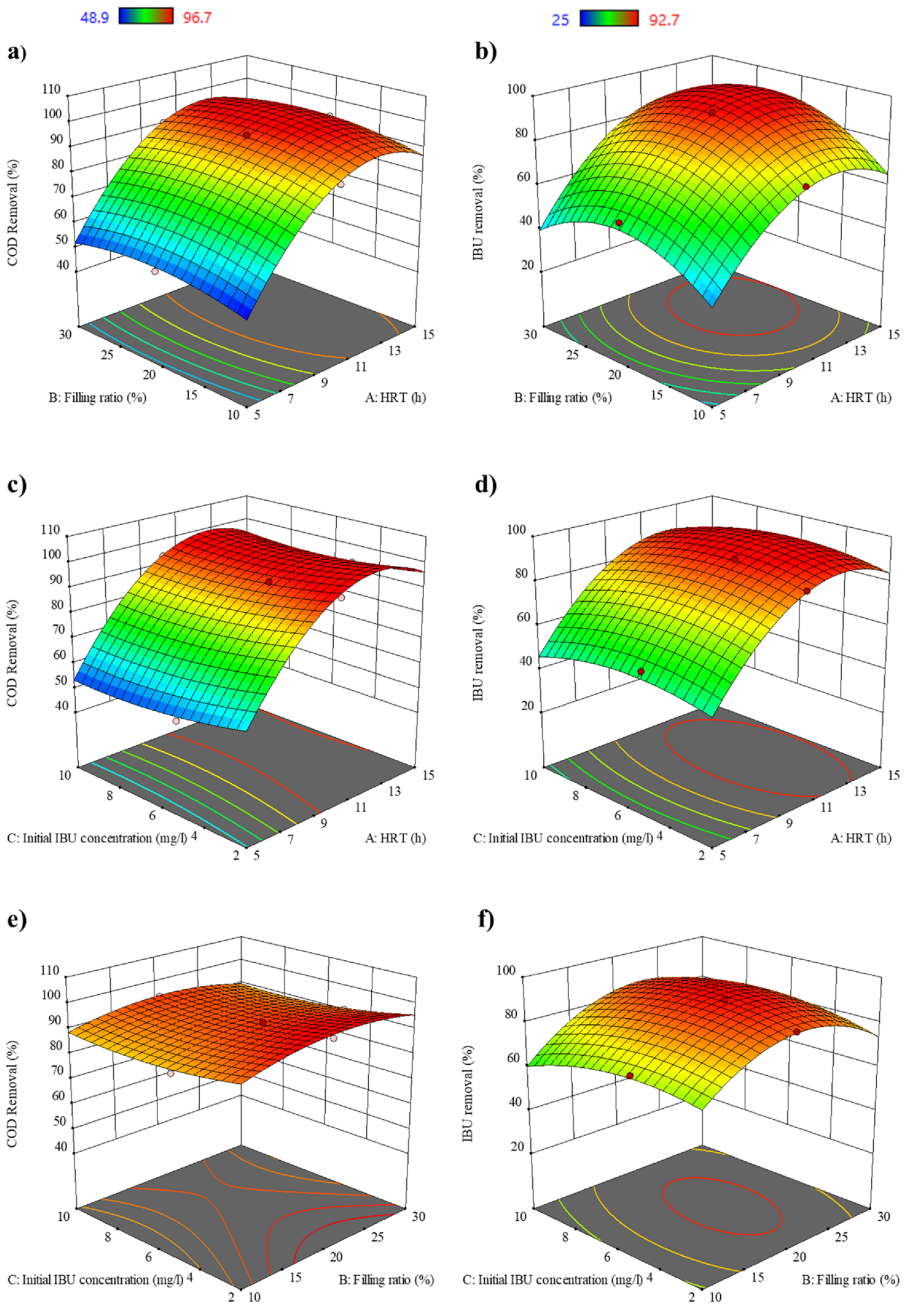
Response surface of COD and IBU removal as a function of (**a**, **b**) HRT and filling ratio, (**c**, **d**) HRT and initial IBU concentration, (**e**, **f**) filling ratio and initial IBU concentration.

The response surface and contour plots in Fig. 11a–f depict the combined impacts of HRT and filling ratio, HRT and initial IBU concentration, and filling ratio and initial IBU concentration on COD and IBU removal. According to Fig. 11a, c, and e, it can be observed that the influence of HRT on COD elimination is significant, and the filling ratio and initial IBU concentration are not significant. In Fig. 11a, it is evident that increasing HRT from 5 to 15 h leads to improvements in COD removal, and increasing the filling ratio from 10 to 30% initially enhances the removal efficiency, followed by a slight decrease. Figure 11c displays a declining trend in COD removal trend as the initial IBU concentration increases. Based on Fig. 11e, it can be found that the COD removal tends to increase with an increase in the filling ratio and a decrease in the initial IBU concentration.

Figure 11b, d, and f show that the HRT and filling ratio have a substantial effect on IBU removal, while initial IBU concentration has a limited effect. Also, in these figures, as the contour plot displays a circle or ellipse, it can be realized that the maximum point is within the experimental region, which is 92.7% for IBU removal^74^. Analyzing Fig. 11b, an increase in HRT causes an overall increase in IBU removal, and the filling ratio initially improves IBU removal but eventually leads to a decrease in its efficiency. Likewise, Fig. 11d illustrates that increasing initial IBU concentration first increases IBU removal and then decreases.

### Optimization of experimental conditions

The parameters were optimized by using the optimization function of Design-Expert software. The optimization process's main objective was to maximize COD and IBU removal and minimize HRT. Minimizing HRT is crucial in some existing WWTPs because they may not have enough space for construction sites^75^. According to the CCD design, the optimum condition was found at HRT = 10 h, filling ratio = 21%, and initial IBU concentration = 3 mg/L for the maximum COD removal of 96.7% and IBU removal of 90.5% from wastewater with a desirability score of 0.837. The desirability function is a widely used multicriteria methodology, converting each response into a function ranging from 0 to 1. If a response meets its target, the function is 1; otherwise, it's 0^74^. In the current study, the desirability score of 0.837, nearing 1, suggests a high level of desirability. At the experimental setting, under conditions of a 10 h HRT, 20% filling ratio, and initial IBU concentrations of 2 mg/L and 5 mg/L, peak COD removal (96.7%) and IBU removal (92.7%) rates were achieved, respectively, closely resembling the results from the CCD design.

## Conclusions

This study investigated the removal of COD and emerging contaminant of IBU from real wastewater of the Southern Tehran WWTP through a sponge-based MBBR process at three different HRTs, filling ratios, and initial IBU concentrations. The results showed that the sponge-based MBBR process could produce highly efficient COD and IBU removal. The peak COD removal rate, reaching 96.7%, was attained under the conditions of an HRT of 10 h, a filling ratio of 20%, and an initial IBU concentration of 2 mg/L. Concurrently, the IBU removal rate, peaking at 92.7%, was observed under the HRT of 10 h, a filling ratio of 20%, and an initial IBU concentration of 5 mg/L. Extending the HRT from 5 to 10 h led to a significant enhancement in the removal of both COD and IBU. However, further extension from 10 to 15 h showed a marginal improvement in the removal efficiency. The optimal filling ratio was determined to be 20% due to the uniform circulation of carriers. Increasing the initial IBU concentration from 2 to 5 mg/L generally improved the removal of COD and IBU. In contrast, an increase from 5 to 10 mg/L decreased the removal of COD and IBU.

MBBR offers a cost-effective solution for upgrading the performance and volumetric treatment capacity of existing wastewater treatment plants, with minimal capital, maintenance, operational, and replacement expenses^52,76^. The estimated cost for treating 1 m^3^ of wastewater with MBBR is approximately US$ 0.15^77^. This study demonstrates the potential of sponge-based MBBR technology for effective COD and IBU removal from wastewater. However, to increase its efficiency, further research is recommended. This includes examining the impact of hospital wastewater to comprehend the combined effects of various pharmaceutical compounds on their removal, evaluating IBU sorption to both sponges and MLSS, and conducting a detailed investigation of the microbial community under various conditions to identify effective microorganisms in IBU removal.

## Data Availability

The datasets generated during and/or analyzed during the current study are included in this published article.

## Abbreviations

MBBR: Moving bed biofilm reactor
COD: Chemical oxygen demand
IBU: Ibuprofen
DO: Dissolved oxygen
MLSS: Liquor suspended solids
MLVSS: Mixed liquor volatile suspended solids
HRT: Hydraulic retention time
CCD: Central composite design
PhAC: Pharmaceutically-active compound
WWTP: Wastewater treatment plants
NSAID: Non-steroidal anti-inflammatory drug
WHO: World Health Organization
MBR: Membrane bioreactor
AOP: Advanced oxidation process
RSM: Response surface methodology
HPLC: High-performance liquid chromatography
PVDF: Polyvinylidene fluoride
ANOVA: Analysis of variance
TOC: Total organic carbon
F/M: Food to microorganism ratio
